# Two‐Dimensional Triferroics: From Fundamental Couplings to Multifunctional Applications

**DOI:** 10.1002/advs.202524376

**Published:** 2026-03-26

**Authors:** Yang Li, Jialin Gong, Zhiqing Li

**Affiliations:** ^1^ Chongqing Higher Education Intelligent Display and Perception Technology Engineering Research Center Chongqing Youth Vocational & Technical College Chongqing China; ^2^ Tianjin Key Laboratory of Low Dimensional Materials Physics and Preparing Technology Department of Physics Tianjin University Tianjin China; ^3^ Institute for Superconducting and Electronic Materials Faculty of Engineering and Information Sciences University of Wollongong Wollongong New South Wales Australia

**Keywords:** ferroelasticity, ferroelectricity, ferrovalley, magnetism, triferroics

## Abstract

Triferroic compounds, characterized by the coexistence and coupling of more than two ferroic orders, have attracted considerable attention as a novel class of multiferroic materials. Compared with conventional multiferroics dominated by pairwise coupling, the involvement of a third ferroic order enables more complex and tunable functional responses, facilitating deterministic multistate control. Although triferroicity has so far been observed only in 3D systems, extending it to 2D materials is particularly appealing, as 2D systems offer advantages such as high storage density, low energy consumption, and mechanical flexibility. In this review, we provide a comprehensive overview of recent progress in 2D triferroic materials, with a particular focus on the origin and interplay of the three ferroic orders at the structural and electronic levels. In addition, we highlight potential applications of 2D triferroics, including multistate storage and spintronic devices. Finally, we outline several promising directions for future research and development in the field of 2D triferroics.

## Graphical abstract

This graphic summarizes the three main types of currently reported 2D triferroic couplings. From the structural perspective, existing systems can be broadly classified into two categories, which exhibit distinct symmetry features and coupling behaviors. Beyond the lattice difference, a third type involves the interplay among ferroelectricity, magnetism, and ferrovalley degrees of freedom. Overall, this figure provides a concise picture of the representative coupling schemes in current 2D triferroic systems and highlights their rich multifunctional responses.

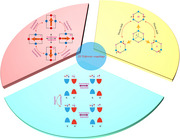

## 1 Introduction

Ferroic orders refer to a class of spontaneous long‐range ordered states [1]. Among them, ferroelectric (FE) order is characterized by spontaneous electric polarization that can be reversed by an external electric field. Magnetic order arises from ordered spin configurations, such as ferromagnetic (FM) or ferrimagnetic (FIM) states, which can be manipulated by magnetic fields. Ferroelastic (FA) order is defined by spontaneous strain and the presence of two or more symmetry‐equivalent orientation variants that can be reversibly switched by external mechanical stress. When two or more ferroic orders are present in a single system, they can give rise to multiferroic behavior, providing multicoupled functionalities that are absent in single ferroic systems. Such materials have attracted significant interest due to their potential for multifunctional applications in sensing and computing [2], nonvolatile memory devices [3], and transistors [4]. At present, multiferroic couplings are generally classified into two main categories, depending on the microscopic origin and the nature of the interactions between different ferroic orders. The first is magnetoelectric coupling, which can be generally categorized into two types. In type‐I multiferroics, ferroelectricity and magnetism arise from different microscopic origins, resulting in only moderate magnetoelectric coupling. Nevertheless, these systems typically exhibit large spontaneous polarization and relatively high ferroic transition temperatures, which make them attractive for practical device applications [5, 6]. In contrast, type‐II multiferroics exhibit ferroelectricity that is directly induced by specific magnetic orders, typically classified into noncollinear spin systems [7, 8, 9, 10, 11] and collinear spin systems [12, 13, 14]. In these materials, the FE polarization is generated directly by the magnetic order, and its intimate intertwining with magnetism gives rise to an intrinsic magnetoelectric coupling. Such coupling allows the manipulation of magnetization by an external electric field [15] or, conversely, the control of polarization by a magnetic field [16]. The second mechanism is magnetoelastic coupling, where magnetic order is directly coupled to lattice strain. In this case, mechanical strain modifies the spin configuration, enabling strain‐mediated control of magnetism and mechanically multifunctional devices [17, 18, 19, 20, 21, 22, 23].

Together, these magnetoelectric and magnetoelastic coupling mechanisms constitute the central framework of conventional multiferroic research, enabling mutual control among different ferroic orders through external electric, magnetic, or mechanical fields. A natural extension of this framework is to explore whether more than two ferroic orders can be simultaneously integrated and coupled within a single material, giving rise to triferroicity and thereby unlocking multifunctional responses beyond those achievable in conventional multiferroic systems. Triferroicity was first realized in 3D materials, with its existence confirmed by both theory [24, 25] and experiment [26, 27]. It is noteworthy that many fundamental physical phenomena initially discovered in 3D materials have subsequently found their counterparts in 2D systems like topological insulators [28, 29, 30], long‐range magnetic ordering [31, 32, 33], and excitonic effects [34, 35]. This naturally motivates extending the search for triferroics to 2D systems. Compared with their 3D counterparts, 2D triferroics offer several distinctive advantages. First, the atomically thin geometry makes their ferroic order parameters and cross‐couplings more susceptible to external fields such as electrostatic gating, strain, thickness variation, and stacking engineering [36]. Second, 2D materials can be readily assembled into van der Waals heterostructures, thereby providing a highly flexible platform for integrating and tailoring multiple ferroic functionalities. Third, reduced dimensionality and symmetry breaking in 2D systems can generate pronounced interfacial and symmetry‐dependent responses, providing favorable conditions for strong ferroic coupling and multistate switching [37]. In this context, 2D triferroics, where three ferroic orders coexist and interact, represent an exciting frontier for realizing novel device concepts and multifunctional platforms. In these systems, FE‐magnetic‐FA or FE‐magnetic‐valley orders are intimately coupled via symmetry‐allowed interactions. For instance, altering one order parameter, such as mechanical strain, can induce a reorientation of magnetic moments and a reversal of the FE polarization. Understanding and classifying these couplings is essential for elucidating the fundamental physics of 2D ferroic systems and for guiding the rational design of nanoscale multifunctional devices.

Despite substantial efforts devoted to the exploration of 2D triferroic materials in recent years, a comprehensive review that systematically summarizes the recent progress and outlines the future directions of this rapidly evolving field is still lacking. Therefore, we provide a timely and comprehensive overview of recent progress in 2D triferroic materials, with a particular focus on the origin and interplay of the three ferroic orders at the atomic and structural levels. In particular, we highlight the potential applications of 2D triferroics in multifunctional devices and outline several promising directions for future research and development in this field.

## 2 Ferroelectricity, Magnetism, and Ferroelasticity Coupling

Building on the above discussion, it is instructive to further examine how 2D triferroic coupling is established in currently proposed systems. 2D triferroic couplings do not arise from a single universal mechanism, but from several representative microscopic routes. In currently reported candidates, such couplings are commonly enabled by lattice distortions, Jahn–Teller effects, charge redistribution, chemical substitution, or interlayer sliding in van der Waals bilayers. It is worth noting that, because the ferroic orders are mutually coupled and influence one another, the specific combination of ferroic orders has a substantial impact on the triferroic coupling. To provide a clear framework for the discussion that follows, we first categorize the triferroic behaviors according to the underlying magnetic orders: FM, antiferromagnetic (AFM), and FIM.

### 2.1 Ferroelectricity, Ferromagnetism, and Ferroelasticity Coupling

The emergence of 2D ferromagnets has provided a versatile foundation for exploring spin‐dependent physics and multifunctional coupling phenomena at the atomic scale [32, 38]. With the rapid progress in 2D magnetism, it is now widely recognized that ferromagnetism alone is often insufficient to unlock the full functional landscape of 2D crystals. This has motivated growing efforts to identify materials where magnetic order is intrinsically entangled with other ferroic responses, enabling correlated manipulation of spin, charge, and lattice degrees of freedom.

**FIGURE 1:**
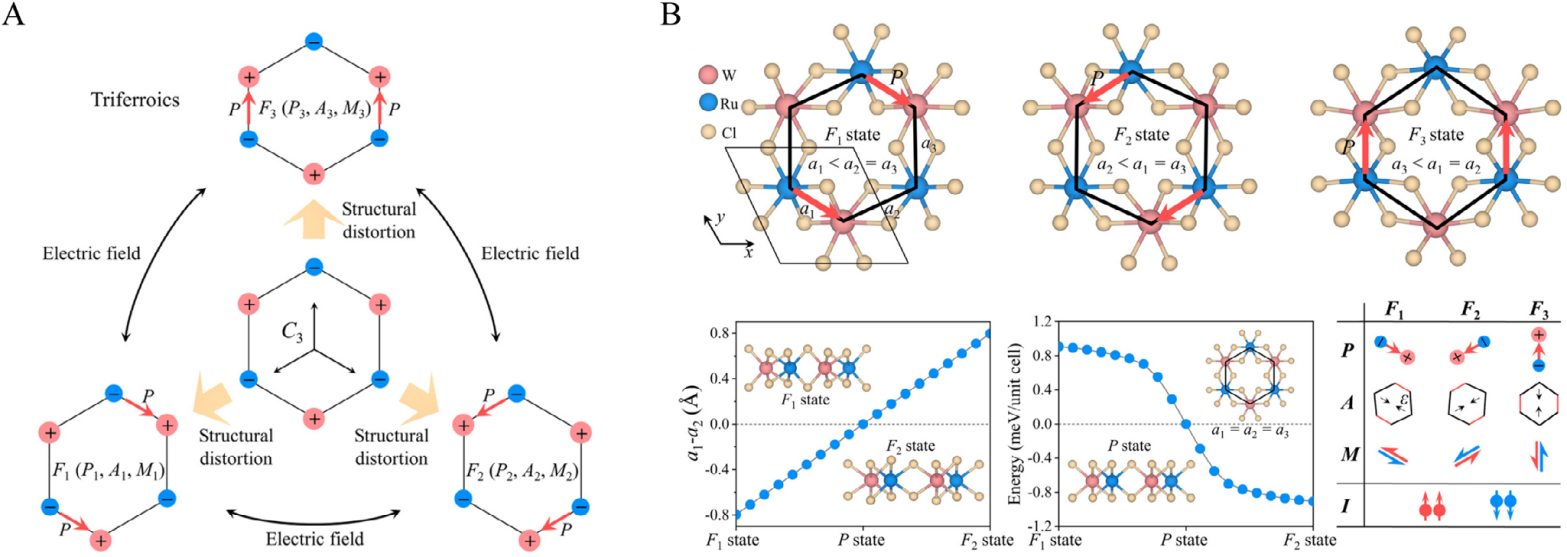
(A) Illustration of electrically controlled triferroic responses in a heteronuclear dual transition‐metal trihalide monolayer. (B) Top view of three symmetry‐equivalent variants of the WRuCl_6_ monolayer. Changes in the lattice distortion (a_1_‐a_2_) and total energy (E) of the WRuCl_6_ monolayer during the F_1_ to F_2_ phase transition. Potential multistate storage behavior in the triferroic WRuCl_6_ monolayer. Reproduced with permission [39]. Copyright 2024, American Physical Society.

Our discussion begins with intrinsic triferroic materials in which FE, FM, and FA orders coexist and are intrinsically coupled. A prototypical material family is the heteronuclear M_1_M_2_X_6_ trihalide monolayers derived from MX_3_: replacing one of the two equivalent metal sublattices breaks inversion symmetry and the C_3_ rotational symmetry, thereby activating a Jahn–Teller–type structural distortion and yielding switchable FE/FA states (Figure 1A). Within this family, Yu et al. [39] identified the dual‐metal trihalide WRuCl_6_ as an intrinsic 2D triferroic candidate that combines 120° rotational ferroelectricity, ferromagnetism, and ferroelasticity. The triferroic nature of this material is driven by spontaneous structural distortions originating from the Jahn‐Teller effect, which triggers the dimerization of neighboring W and Ru atoms. This structural distortion directly dictates the electronic and magnetic properties of the system. Microscopically, the distorted octahedral coordination splits the transition‐metal d orbitals into t_2g_ and e_g_ states, yielding a sizable moment on W (1.808 µB) while Ru remains nearly zero (0.017 µB), consistent with Hund's rule and the Pauli exclusion principle.

As shown in Figure 1B, three FE states of the WRuCl_6_ monolayer correspond to three FA orientation variants, arising from three equivalent distortion directions of the paraelectric (PE) phase. Consequently, the FE and FA orders are intrinsically intertwined. The transition between different FA states is accompanied by the reorientation of the electric polarization, which effectively enables electrically controlled ferroelasticity. Notably, this triferroic coupling also drives a reorientation of the magnetic easy axis, allowing electrically controlled magnetism in the WRuCl_6_ monolayer. Beyond those FA/FE/FM orders, the WRuCl_6_ monolayer exhibits spin‐polarized carriers (I_1_ and I_2_) as additional storage parameters, thereby providing distinct signals to improve the storage capacity. This coexistence of robust triferroic coupling and multiple storage states making WRuCl_6_ monolayer a promising platform for high‐density, multistate information storage and multifunctional nanodevices.

**FIGURE 2:**
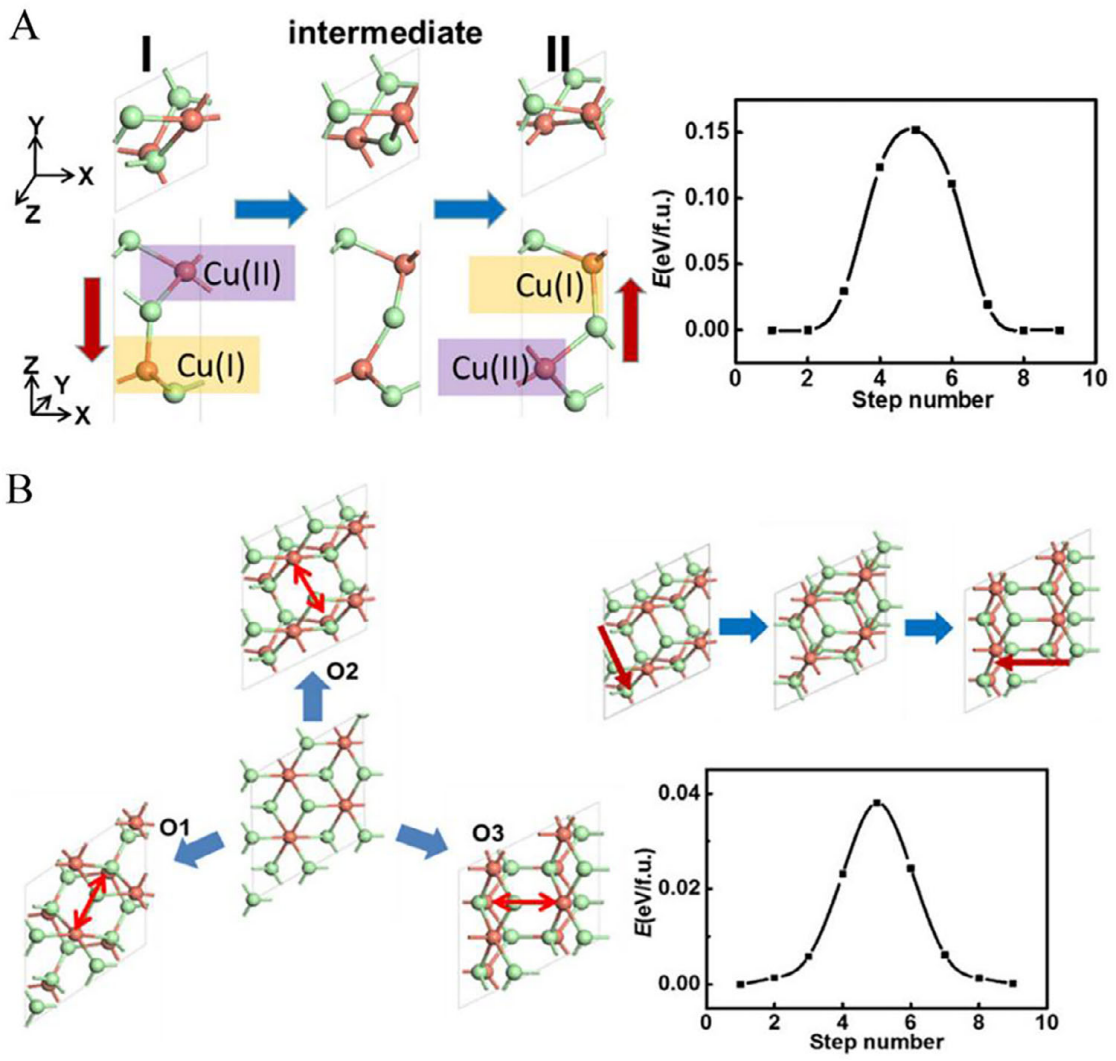
(A) Vertical FE switching of Cu_2_Cl_3_ and the corresponding computed switching pathway. (B) Three symmetry‐equivalent in‐plane orientation states and a computed switching pathway. Reproduced with permission [40]. Copyright 2023, Royal Society of Chemistry.

Another representative example is the mixed‐valence monolayer Cu_2_Cl_3_ proposed by Gao et al. [40] The monolayer Cu_2_Cl_3_ undergoes a spontaneous valence stratification, in which Cu ions segregate into two chemically distinct atomic layers, Cu(I) and Cu(II). This stratification breaks the symmetry and gives rise to a pronounced vertical FE polarization (Figure 2A). Meanwhile, the ferromagnetism in Cu_2_Cl_3_ arises from the Cu(I) ion transferring partial charge to the surrounding Cl network and thus Cu(I) ions are in Cu^1+δ^ state, leading to finite magnetic moments. During the vertical FE switching, the magnetic moments associated with Cu(I) and Cu(II) ions are simultaneously rearranged. Consequently, the FE switching directly drives a magnetic reconfiguration, establishing a strong FE–FM coupling.

Crucially, in addition to the vertical ferroelectricity, monolayer Cu_2_Cl_3_ develops independent in‐plane FE and FA orders originating from the Jahn‐Teller distortion of the Cu ions. This distortion produces in‐plane polarization and a spontaneous anisotropic strain, which leads to three symmetry‐equivalent orientation variants (O1, O2, and O3), thereby establishing an intimate coupling between FA strain and in‐plane FE polarization (Figure 2B). Because the out‐of‐plane and in‐plane ferroelectricity arise from fundamentally different microscopic mechanisms, their coexistence yields a total of six equivalent states, thus enabling a multistate landscape with enhanced tunability. In conclusion, monolayer Cu_2_Cl_3_ integrates vertical FE‐FM coupling and independent in‐plane FE‐FA coupling, offering new perspectives for the rational design of 2D triferroic materials.

**FIGURE 3:**
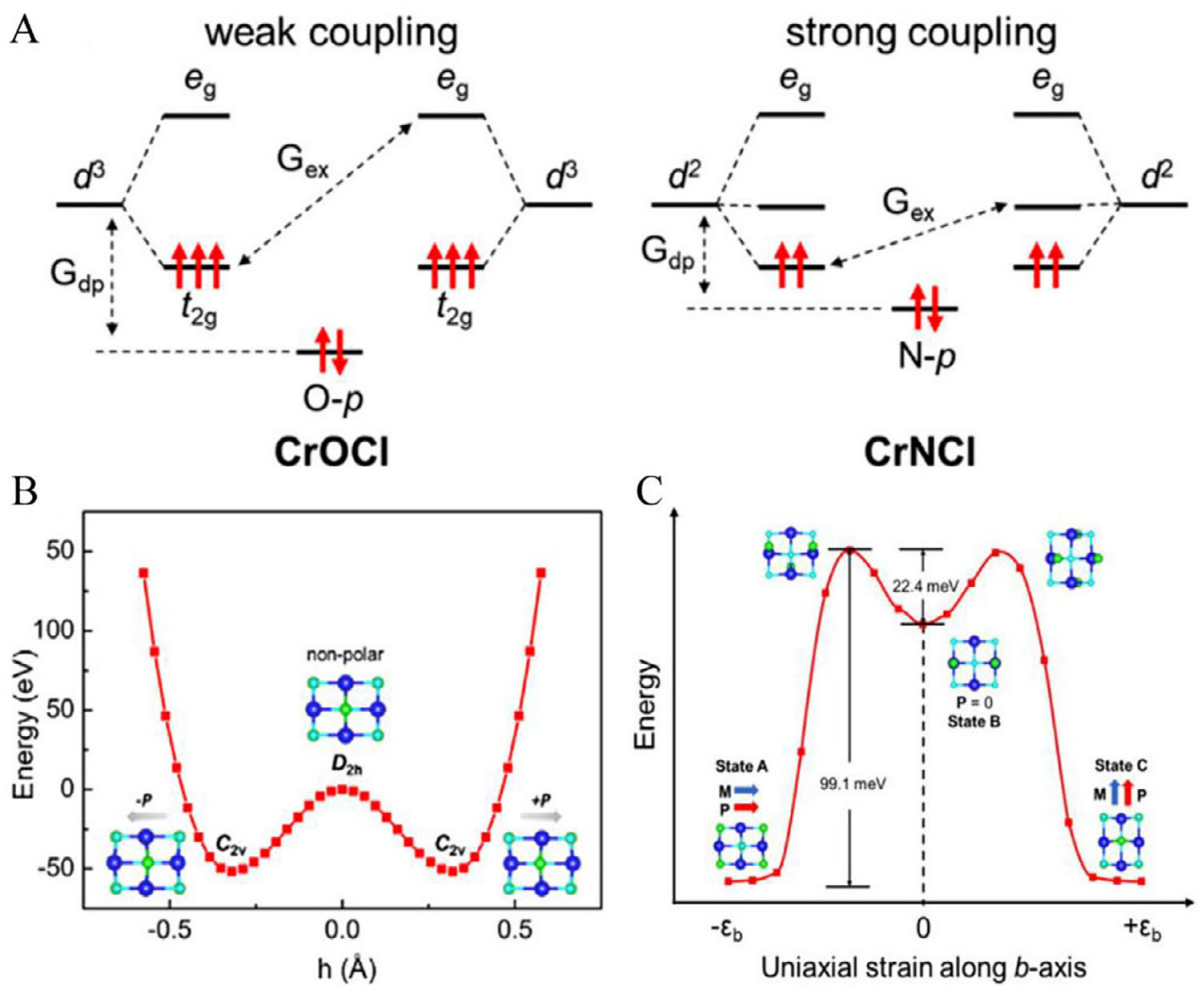
(A) virtual exchange gap (G_ex_) and d‐p hybridization gap (G_dp_) in CrOCl and CrNCl monolayers. (B) Relative energy change with respect to the structural parameter h and switching pathway for the in‐plane polarization reversal. (C) Strain‐induced reorientation of in‐plane electric polarization and magnetization. Reproduced with permission [45]. Copyright 2023, American Institute of Physics.

It's worth noting that the working temperatures of most existing multiferroic materials remain far below room temperature, which severely limits their practical applications. This limitation mainly originates from the difficulty of stabilizing multiple ferroic orders and their mutual coupling within the same temperature range. In many multiferroics, the FE and magnetic transition temperatures do not coincide, and in a large number of cases, FE order appears at considerably higher temperatures than magnetic ordering. Moreover, the simultaneous coexistence of ferroelectricity, magnetic ordering, and ferroelasticity in a single phase generally requires the coordinated stabilization of lattice, charge, spin, and strain, which are often governed by different or even competing microscopic interactions. As a result, the corresponding multifunctional states are usually stabilized only within a limited low‐temperature window [41, 42, 43, 44]. Consequently, the pursuit of room‐temperature intrinsic triferroic materials has emerged as an important direction in the field. Sun et al. [45] reported that the CrNCl monolayer is a potential 2D triferroic semiconductor at room temperature. The *d*–*p* hybridization between Cr and N drives a spontaneous Cr–N dimerization, therefore breaking inversion symmetry and producing a robust in‐plane polarization (Figure 3B). Simultaneously, as shown in Figure 3A, the structural distortion further splits the Cr *t_2g_* ↑ level so that two *d* electrons occupy the lower *d_xz,xy_* levels while the *d_yz_* level remains empty, resulting in a reduced virtual exchange gap G_ex_ compared to CrOCl. Together with the smaller electronegativity of N, the N‐p levels are higher in energy than the O‐p levels, which results in a smaller G_dp_ and stronger *d*‐*p* hybridizations; these electronic features significantly enhance the FM superexchange interactions, leading to a comparatively high Curie temperature *T_c_* of ∼760K. The strain‐mediated magnetoelectric response of the CrNCl monolayer arises when uniaxial strain is applied along the in‐plane direction. Specifically, during the transition from a FE–FM state (A) to a degenerate FE–FM state (C), FA switching induces a 90° rotation of the polarization and magnetization, thereby revealing a strong FA–magnetoelectric coupling (Figure 3C). Consequently, the CrNCl monolayer serves as a rare example of a room‐temperature triferroic candidate, providing a practical blueprint for functional 2D triferroic nanodevices.

However, intrinsic triferroic systems remain rare, highlighting the importance of exploring strategies to induce triferroicity within conventional 2D materials. Among them, doping has emerged as a powerful strategy to tailor the electronic, magnetic, and ferroic properties of 2D materials [46, 47, 48, 49]. Bhardwaj et al. [50] achieved hole‐doped GdCl_2_ monolayer via partial substitution of Gd^2^^+^ with Eu^2^^+^. Upon hole doping, the system develops a bond‐centered charge ordering accompanied by cooperative lattice distortions, resulting in spontaneous polarization and strain. Due to the charge ordered state resembling the “Zener polaron order” [51, 52], all spins are expected to align coherently, producing a FM ground state. Moreover, based on the calculation of magnetoanisotropy energy and exchange parameters, the resulting FM state is expected to exhibit a transition temperature comparable to that of the pristine GdCl_2_ monolayer (∼224 K).

**FIGURE 4:**
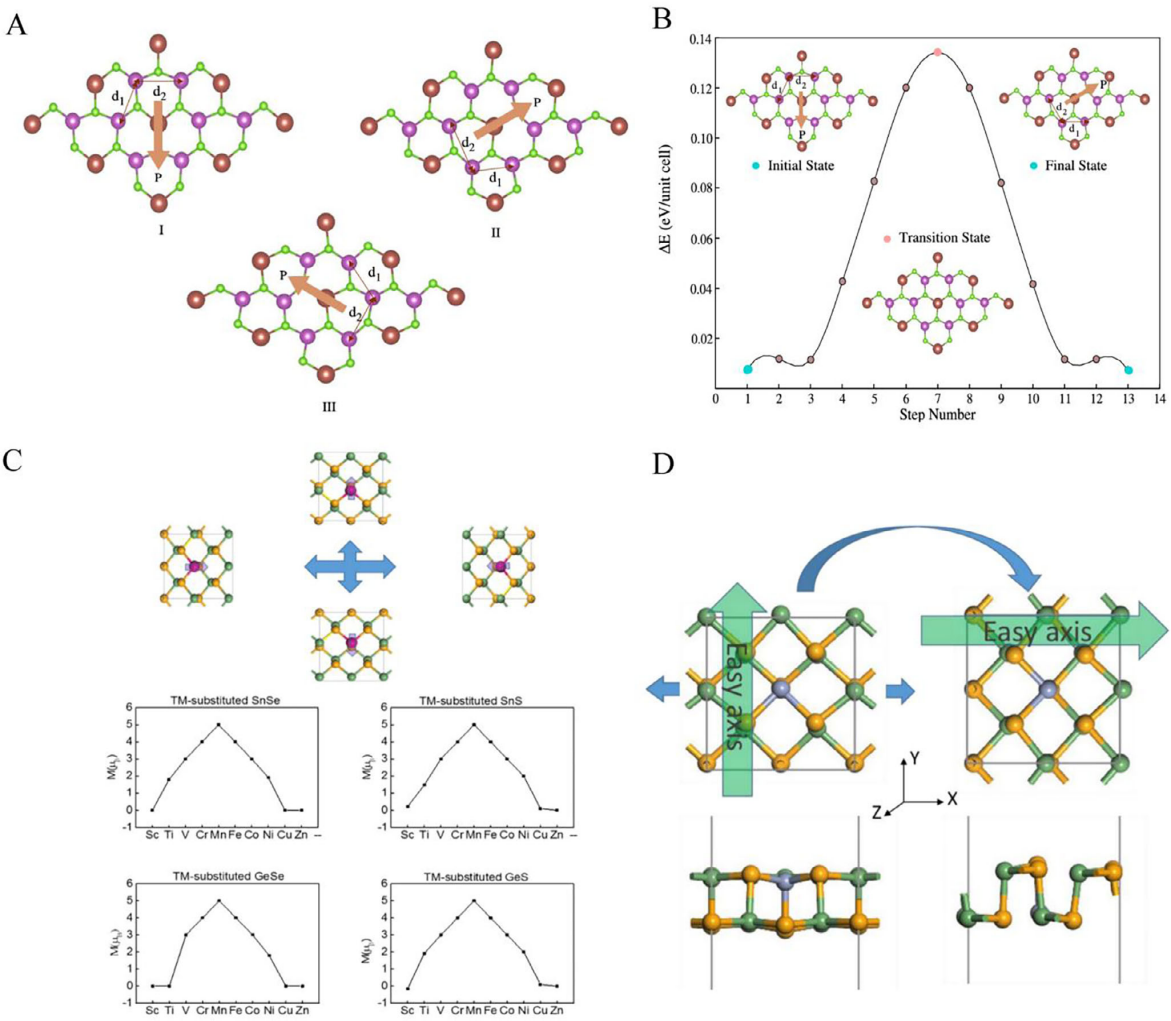
(A) Spontaneous polarization directions of Eu‐substituted GdCl_2_ monolayer in different lattice configurations. (B) Transition pathway between two FA phases. Reproduced with permission [50]. Copyright 2023, American Physical Society. (C) TM‐doped group‐IV monochalcogenide (SnS, SnSe, GeS, GeSe) monolayers and their related magnetic moment. (D) Switching of the magnetic easy axis by an external electric field or strain. Reproduced with permission [54]. Copyright 2018, Institute of Physics.

Given that the undistorted structure is hexagonal with threefold rotational symmetry, the lattice distortion can be accommodated along any one of these three symmetry‐related directions, giving rise to three energetically degenerate FA (FE) states as shown in Figure 4A. Furthermore, since the spontaneous polarization is inherently tied to the orientation of the charge‐ordering state and the lattice distortion, reorienting the strain direction will directly switch the polarization direction. This feature indicates that, in the Eu‐substituted GdCl_2_ monolayer, the FA (FE) switching processes share a common switching pathway, and the corresponding minimum‐energy transition barrier between two FA (FE) phases is shown in Figure 4B. In summary, the Eu‐substituted GdCl_2_ monolayer exhibits strong coupling between ferroic orders, making it a highly versatile candidate for multifunctional device applications.

Group‐IV monochalcogenides, including SnS, SnSe, GeS, and GeSe, have been predicted to behave as a 2D FA–FE multiferroic with giant spontaneous polarization and intrinsic lattice strain [53]. However, the absence of intrinsic magnetoelectric coupling significantly constrains their functional applicability. Accordingly, Yang et al. [54] proposed that doping 3D transition‐metal ions into these monolayers can introduce localized magnetic moments, thereby enabling triferroic coupling, as illustrated in Figure 4C. Specifically, when the magnetic easy axis initially lies along the x direction, it rotates to the y axis under either a FE switching or a 90° FA rotation, as shown in Figure 4D. Thus, doped group‐IV monochalcogenides provide a chemically tunable platform for achieving 2D triferroicity, enabling efficient non‐volatile data reading and writing.

**FIGURE 5:**
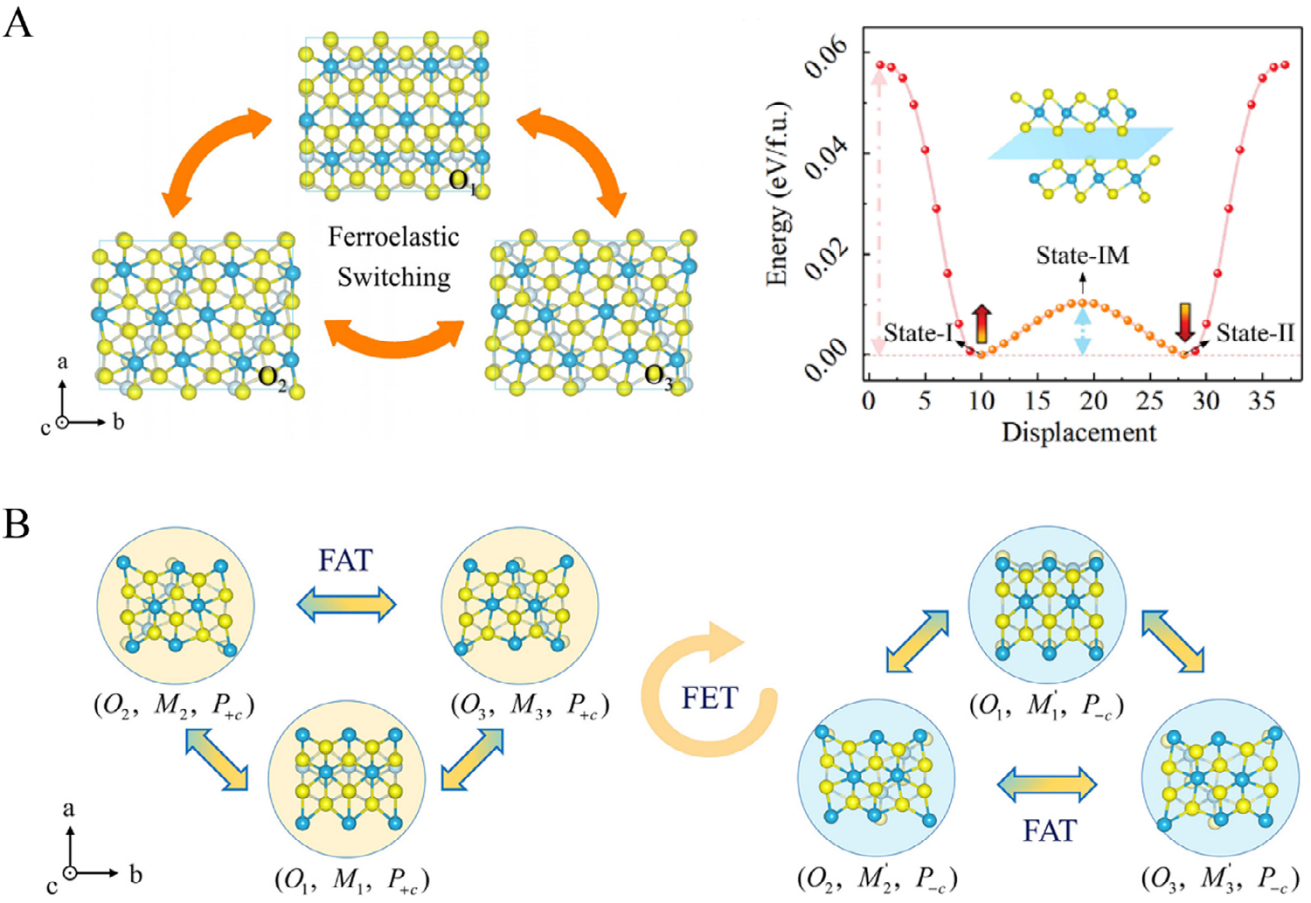
(A) FA and FE transition pathways of T'‐VTe_2_. (B) Mechanism of the six‐state triferroicity of bilayer T'‐VTe_2_. Reproduced with permission [60]. Copyright 2022, American Institute of Physics.

Single‐phase multiferroics, particularly in the 2D lattice, remain scarce due to the fundamental incompatibility between the electronic and symmetry requirements of different ferroic orders. To address these challenges, sliding ferroelectricity in van der Waals (vdW) bilayers has emerged as a promising strategy. It originates from the interlayer charge transfer and layer‐relative ionic shifts [55, 56, 57, 58], thereby avoiding the conventional incompatibility with ferromagnetism. Furthermore, FA order is typically compatible with standard vdW stacking arrangements [59], thereby overcoming the fundamental restrictions imposed by symmetry. Zhang et al. [60] proposed a candidate bilayer T′‐VTe_2_ which exhibits ferromagnetism, ferroelasticity, and sliding ferroelectricity simultaneously. The FM order in bilayer T′‐VTe_2_ arises from the combined contributions of intralayer and interlayer FM exchange interactions. Through analysis of the structure of bilayer T′‐VTe_2_, three symmetry‐equivalent phases (*O*_1_, *O*_2_, and *O*_3_) are identified, which can be transformed into one another through a 120° rotational symmetry operation (Figure 5A). Given these characteristics, FA switching in bilayer T′‐VTe_2_ induces a 120° rotation of the lattice, which in turn drives a corresponding 120° rotation of the magnetization easy axis. Furthermore, the FE and FM orders are also strongly coupled because the out‐of‐plane polarization in state‐I points downward, the spin density becomes slightly imbalanced between the two layers, with the down layer carrying a marginally larger magnetic moment. Reversing the polarization swaps this imbalance, allowing FE control over the nonequivalent interlayer magnetic moments and thereby realizing an unusual 2D magnetoelectric coupling.

Therefore, based on the sliding‐driven triferroic mechanism of bilayer T′‐VTe_2_, it offers six logic states as shown in Figure 5B. The FM state is denoted as ***M***, the three FA variants are denoted as ***O_1_***–***O_3_***, and the two FE states with opposite out‐of‐plane polarizations are denoted as ***P_+__c_*** and ***P*** *_−c_*. For a system with ***P_+__c_***, the coupling between the magnetization easy axis and ferroelasticity yields three distinct FM configurations (***M_1_***–***M_3_***), thereby leading to three logic states, labeled as (***O_1_***, ***M_1_***, ***P_+c_***), (***O_2_***, ***M_2_***, ***P_+c_***), (***O_3_***, ***M_3_***, ***P_+c_***). All three states are linked by FA switching. When reversing electric polarization to ***P_−__c_***, because of the magnetoelectric coupling, the original FE states ***M_1_***–***M_3_*** are transformed into ***M_1_^'^***–***M_3_^'^***, characterized by an out‐of‐plane magnetic moment opposite to that of ***M_i_***, so these three states transform into a new set of three logic states (***O_1_***, ***M_1_^'^***, ***P_−__c_***), (***O_2_***, ***M_2_^'^***, ***P_−__c_***), (***O_3_***, ***M_3_^'^***, ***P_−__c_***). The transformation of three states can also be achieved via FA switching. This makes bilayer T′‐VTe_2_ a promising platform for nonvolatile multistate memory and electrically spintronic devices.

### 2.2 Ferroelectricity, Antiferromagnetism, and Ferroelasticity Coupling

AFM materials have attracted extensive attention owing to their absence of stray fields [61], strong immunity to external magnetic perturbations [62], and ultrafast spin dynamics that can reach the terahertz regime [63]. Integrating the antiferromagnetism with ferroelasticity and ferroelectricity enables diverse coupling mechanisms, thereby significantly enriching the functional landscape of 2D triferroics.

**FIGURE 6:**
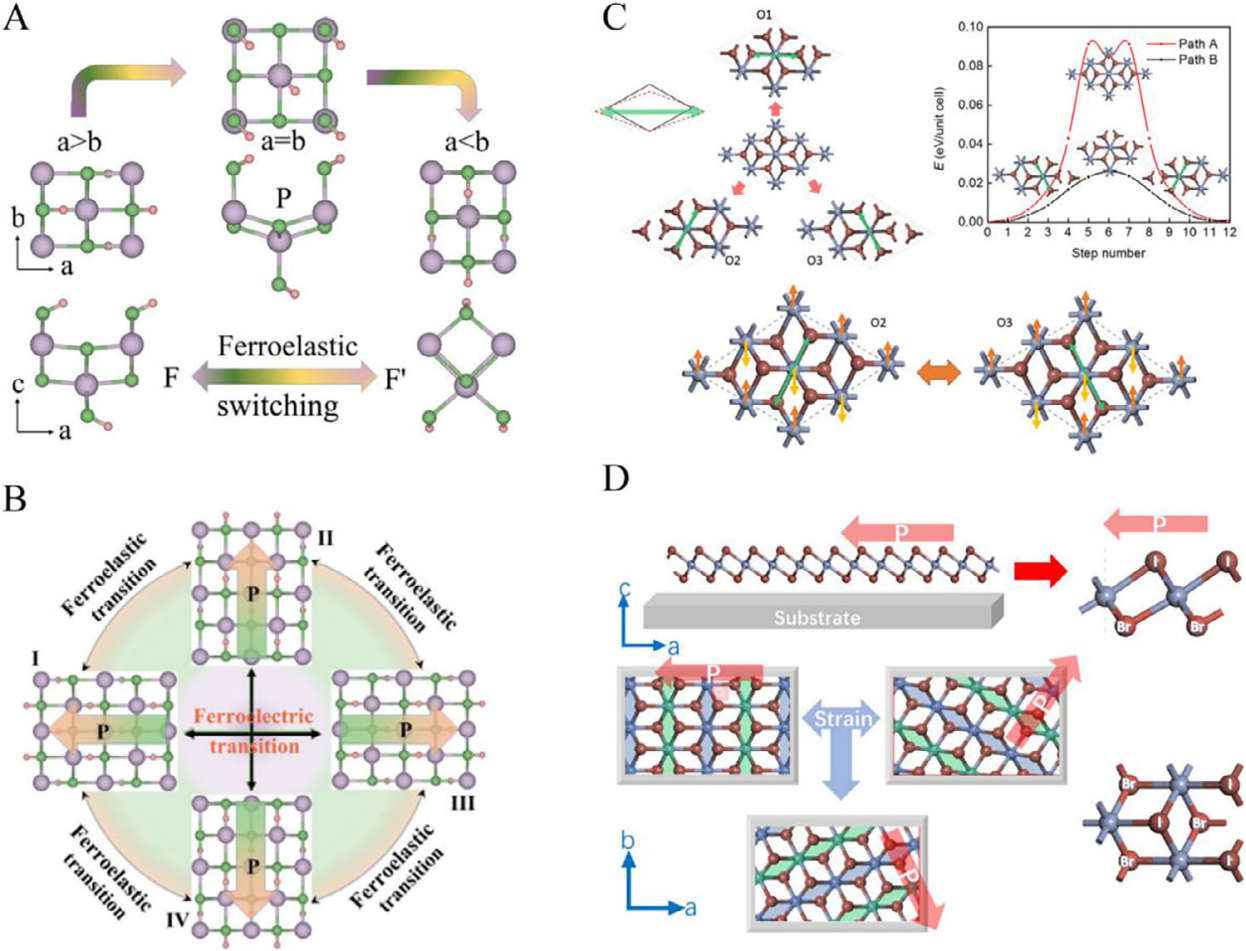
(A) FA pathway of SL FeO_2_H. (B) Triferroic transition pathway of SL FeO_2_H. Reproduced with permission [64]. Copyright 2021, American Physical Society. (C) Three equivalent FA phases and the FA switching pathway of the CrI_2_ monolayer. Evolution of spin configurations driven by FA switching. (D) The in‐plane polarization induced by the substrate and its switching pathway. Reproduced with permission [65]. Copyright 2022, American Physical Society.

Shen et al. [64]. proposed single‐layer (SL) FeO_2_H as a rare example that combines antiferromagnetism, ferroelasticity, and ferroelectricity simultaneously. In this material, the edge‐sharing octahedra with nearly 90° Fe─O─Fe bonds favor FM superexchange, whereas the corner‐sharing geometry with nearly 180° Fe─O─Fe bonds provides a much stronger AFM superexchange, which ultimately drives the system into an AFM ground state. Figure 6A illustrates that SL FeO_2_H hosts two FA variants, F and F′, which are related by a 90° rotation and can be reversibly switched with each other through uniaxial strain. Meanwhile, because the O─H bond deviates from the c‐axis of SL FeO_2_H and thereby breaks mirror symmetry, such a displacement is expected to induce a spontaneous polarization.

As illustrated in Figure 6B, FA switching induces a 90° rotation of the SL FeO_2_H lattice, which in turn results in a corresponding 90° rotation of the polarization ***P_s_***. Building upon this feature, a four‐state memory device can be realized. Specifically, the polarization ***P_s_*** can adopt four directions, which are switchable through FE and FA transitions. Since each FA state is tied to two polarization directions and can be electrically distinguished, the system combines the advantages of FE and FA memory storage. Moreover, because the AFM easy axis aligns along the in‐plane direction, it undergoes a 90° rotation by FA switching, enabling directional control of the easy axis of antiferromagnetism. In summary, SL FeO_2_H is an intrinsic 2D triferroic semiconductor that permits mechanical control of both polarization and spin orientation, offering a promising platform for multi‐level and reconfigurable memory applications.

Following this line, Yang et al. [65] identified monolayer chromium dihalides (CrI_2_) as a promising platform for triferroicity. The magnetic ground state of CrI_2_ adopts a striped AFM configuration, characterized by FM spin chains perpendicular to the Jahn‐Teller (J‐T) elongation, while the interchain coupling behaves AFM. Driven by the J‐T effect, the initially undistorted lattice lowers its energy by elongating along one of the three equivalent directions, enabling FA switching through a 120° rotation of the spontaneous strain (Figure 6C). Moreover, when the J‐T elongated direction rotates by 120° during FA switching, the spin configuration concurrently rotates by 120°, which demonstrates that the magnetism in CrI_2_ is intrinsically coupled to its ferroelasticity.

However, both the undistorted and distorted structures remain centrosymmetric, and thus no electric polarization exists or changes during FA switching. To overcome this limitation, introducing substrate interaction can break the equivalence between the top and bottom I atoms, which may induce polarization in the system. At this stage, the charges of the top and bottom I atoms would be different, but vertical polarization cannot be reversed. On the contrary, the in‐plane polarization is aligned with the elongation direction and can be rotated by 120° under an electric field or via FA switching. Correspondingly, the spin‐stripe orientation also rotates by 120°, indicating that FE, FA, and magnetic orders are mutually coupled as shown in Figure 6D. Such triferroic coupling enables magnetic states to be manipulated through FE/FA switching, thus offering a promising route toward electric‐writing and magnetic‐reading functionalities.

**FIGURE 7:**
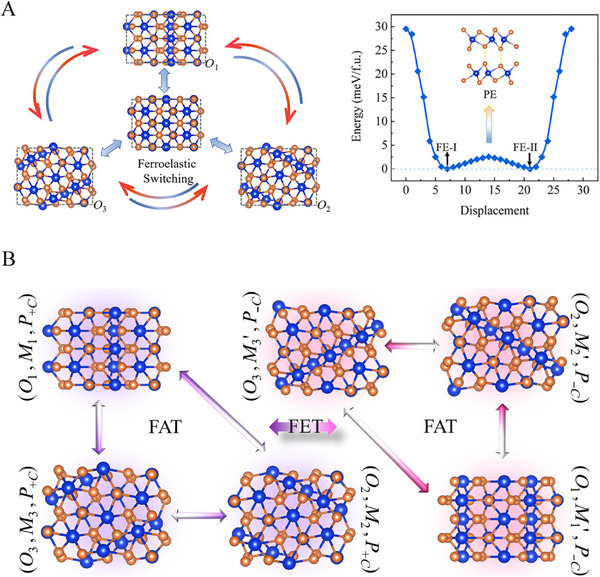
(A) FA and FE switching pathways of bilayer T'‐TiBr_2_. (B) Illustration of switching between six‐states in bilayer T'‐TiBr_2_. Reproduced with permission [66]. Copyright 2024, American Physical Society.

Another example is the bilayer T'‐TiBr_2_, proposed by Chai et al. [66] that exhibits semiconducting triferroicity. Its magnetism originates from a distorted octahedral structure, where two electrons of the Ti atoms partially occupy the *t_2g_* orbitals, producing a local moment of 2 *µ_B_*. Interlayer exchange interactions further stabilize a collinear AFM ground state over the FM configuration. Ferroelasticity arises from dimerization of Ti atoms along three equivalent directions, giving rise to three FA states (***O_1_***, ***O_2_***, and ***O_3_***) whose lattice orientations are rotated by 120° with respect to one another, as shown in Figure 7A. Finally, out‐of‐plane ferroelectricity emerges in the bilayer because the stacking operation breaks inversion symmetry and generates inequivalent charge centers between the two layers.

As a result, unconventional triferroic coupling can be realized in T'‐TiBr_2_. For instance, bilayer T'‐TiBr_2_ exhibits magnetic anisotropy, with its easy axis lying in‐plane at 60° from the a axis. When the lattice orientation switches during the FA transition, the magnetization direction follows accordingly, demonstrating a strong coupling between magnetism and ferroelasticity. Beyond ferroelasticity, switching the FE polarization reverses the spin density distributions of the two layers, resulting in a strong magnetoelectric coupling in bilayer T'‐TiBr_2_. Taken together, these coupled ferroic properties enable bilayer T'‐TiBr_2_ to host six distinct logical states which can be interconverted through FA transitions (FATs) and FE transitions (FETs) as shown in Figure 7B. In detail, the three AFM configurations corresponding to three FA variants, which are denoted as ***M_1_***–***M_3_*** in the ***P_+c_*** state and as ***M_1_*** '–***M_3_*** ' in the ***P_−__c_*** state. Consequently, the system forms six logical states—(***O_1_***, ***M_1_***, ***P_+c_***), (***O_2_***, ***M_2_***, ***P_+c_***), (***O_3_***, ***M_3_***, ***P_+c_***), (***O_1_***, ***M_1_*** ', ***P_−__c_***), (***O_1_***, ***M_2_*** ', ***P_−__c_***), and (***O_1_***, ***M_3_*** ', ***P_−__c_***). In summary, bilayer T'‐TiBr_2_ exhibits triferroicity induced by van der Waals stacking, where FE–FA–AFM orders are strongly coupled. These intertwined ferroic degrees of freedom strongly motivate exploration of triferroic multiferroics.

### 2.3 Ferroelectricity, Ferrimagnetism, and Ferroelasticity Coupling

Among the limited pool of 2D multiferroic materials, the realization of triferroic coupling remains extremely rare, especially at room temperature. This is largely due to the competing nature of d‐electrons in magnetic and FE orders, and the suppression of long‐range ferroic orders in reduced dimensions [67]. In a recent work, Tang et al. [68] proposed a novel strategy to overcome these limitations by introducing electronic asymmetry into FA magnetic lattices. Their design successfully realizes room‐temperature triferroicity in a class of dual‐transition‐metal dichalcogenide monolayers, with 1T′‐CrCoS_4_ serving as a prototype material.

**FIGURE 8:**
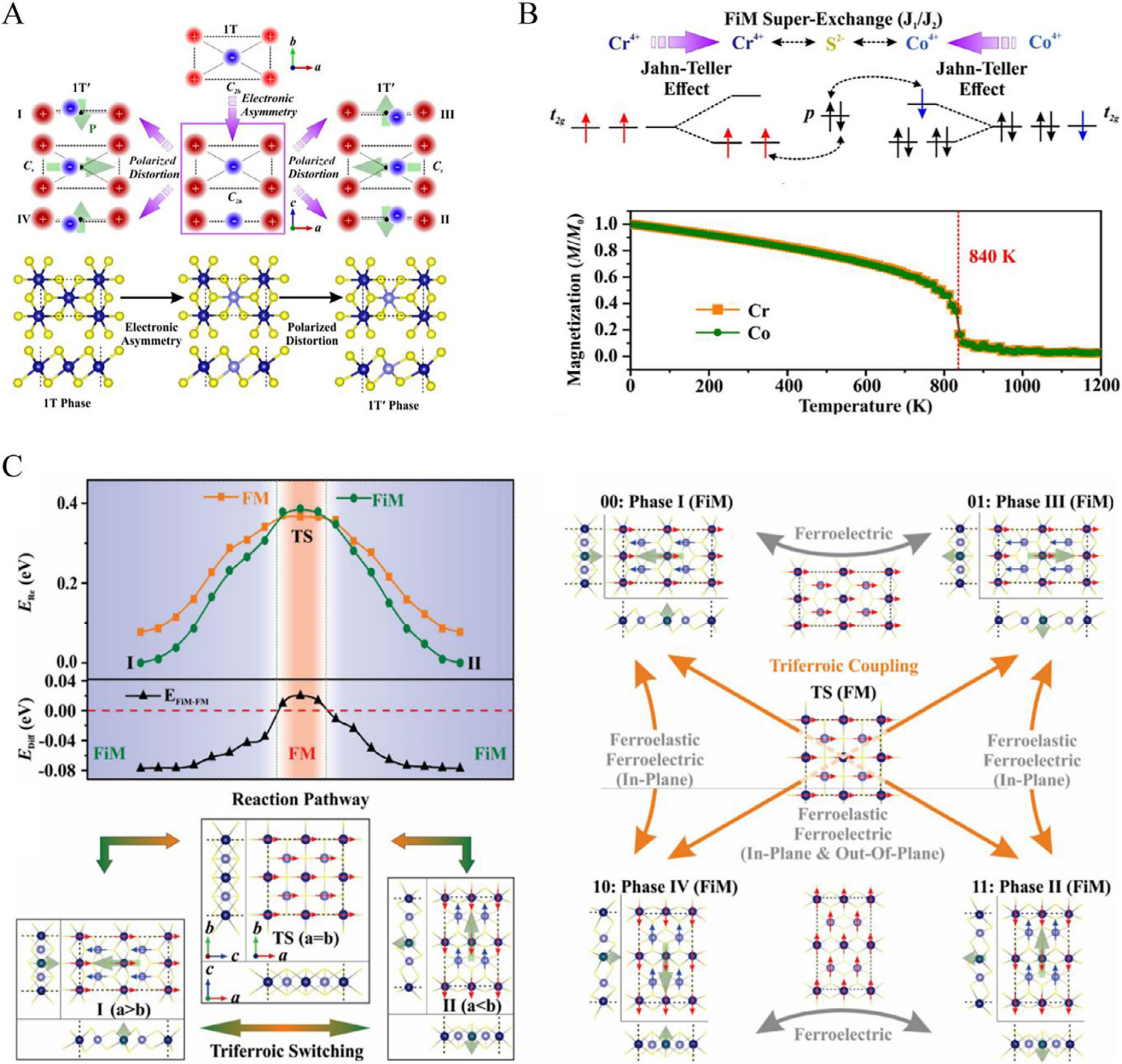
(A) Schematic illustration of the emergence of electric polarization in a 2D tetrahedral lattice. (B) S‐mediated superexchange between Cr^4+^ and Co^4+^ ions. (C) Structural transformation from the centrosymmetric 1T phase to the polar 1T′ phase in dual‐transition‐metal dichalcogenide (TMD) lattices. Reproduced with permission [68]. Copyright 2023, American Chemical Society.

The 1T′‐CrCoS_4_ monolayer exhibits triferroic behavior due to its carefully engineered structure and electronic configuration. The coexistence of Cr and Co atoms introduces electronic asymmetry into the crystal lattice, breaking inversion symmetry and inducing spontaneous electric polarization—thus giving rise to ferroelectricity (Figure 8A). This distortion lowers the crystal symmetry to the *C_s_* symmetry, which further allows for the emergence of both in‐plane and out‐of‐plane polarization. FIM arises from the antiparallel alignment of Cr and Co magnetic moments, mediated through a superexchange interaction via sulfur (S) atoms, which results in a net magnetic moment of 1 µ_B_ per unit cell and a Curie temperature of up to 840 K, well above room temperature (Figure 8B). Ferroelasticity, on the other hand, is driven by the anisotropic lattice structure. The material can reversibly switch under mechanical strain, with a calculated energy barrier of 0.37 eV/atom and a reversible strain magnitude of 66.7%. Together, these three ferroic orders form the foundation of the triferroic behavior in 1T′‐CrCoS_4_ monolayer.

What distinguishes 1T′‐CrCoS_4_ is not only the presence of three ferroic orders, but also the strong intrinsic coupling among them. During FA and FE switching, the material undergoes a FIM‐FM‐FIM magnetic transition, indicating that magnetism is directly modulated by strain and electric field. This feature results in four FA‐FE states (00, 01, 10, 11) as shown in Figure 8C. Meanwhile, the magnetic anisotropy of the easy‐plane FIM state is switchable between the *x* and *y* directions. Consequently, the in‐plane polarization can be deterministically reoriented among *x*, −*x*, *y*, and −*y*, while the out‐of‐plane polarization can be reversed between *z* and −*z*. This combined control enables information to be written and read in eight distinct states (e.g., *xz, x–z, yz, y‐z, ‐xz, ‐x‐z, –yz*, and *–y–z*), thereby offering a mechanically, electrically, and magnetically addressable multistate nanodevice platform.

### 2.4 Antiferroelectricity, Altermagnetism, and Ferroelasticity Coupling

Altermagnetism has recently been recognized as a distinct form of collinear magnetism, with zero net magnetization, in which opposite spin sublattices are related by crystal‐rotation symmetries [69]. This symmetry‐imposed magnetism produces momentum‐dependent alternating spin splitting, giving rise to large anomalous Hall effects [70], spin‐polarized transport [71], and unconventional responses previously considered exclusive to FMs [72]. In parallel, antiferroelectricity has emerged as an important antipolar structural order, characterized by reversible switching between degenerate antipolar states through an intermediate polar phase [73]. Such switching pathways enable large energy‐storage densities [74], and non‐volatile functional responses that complement those found in ferroelectrics [75]. Recent studies have begun to explore the coexistence and coupling between altermagnetism and antiferroelectricity, revealing the possibility of electrically switchable spin states, antipolar‐controlled spin splitting, and new classes of ferroic‐derived multifunctional materials [76, 77, 78]. These emerging directions highlight the rich opportunities at the intersection of AM and antiferroelectric (AFE) orders for designing next‐generation spintronic devices.

**FIGURE 9:**
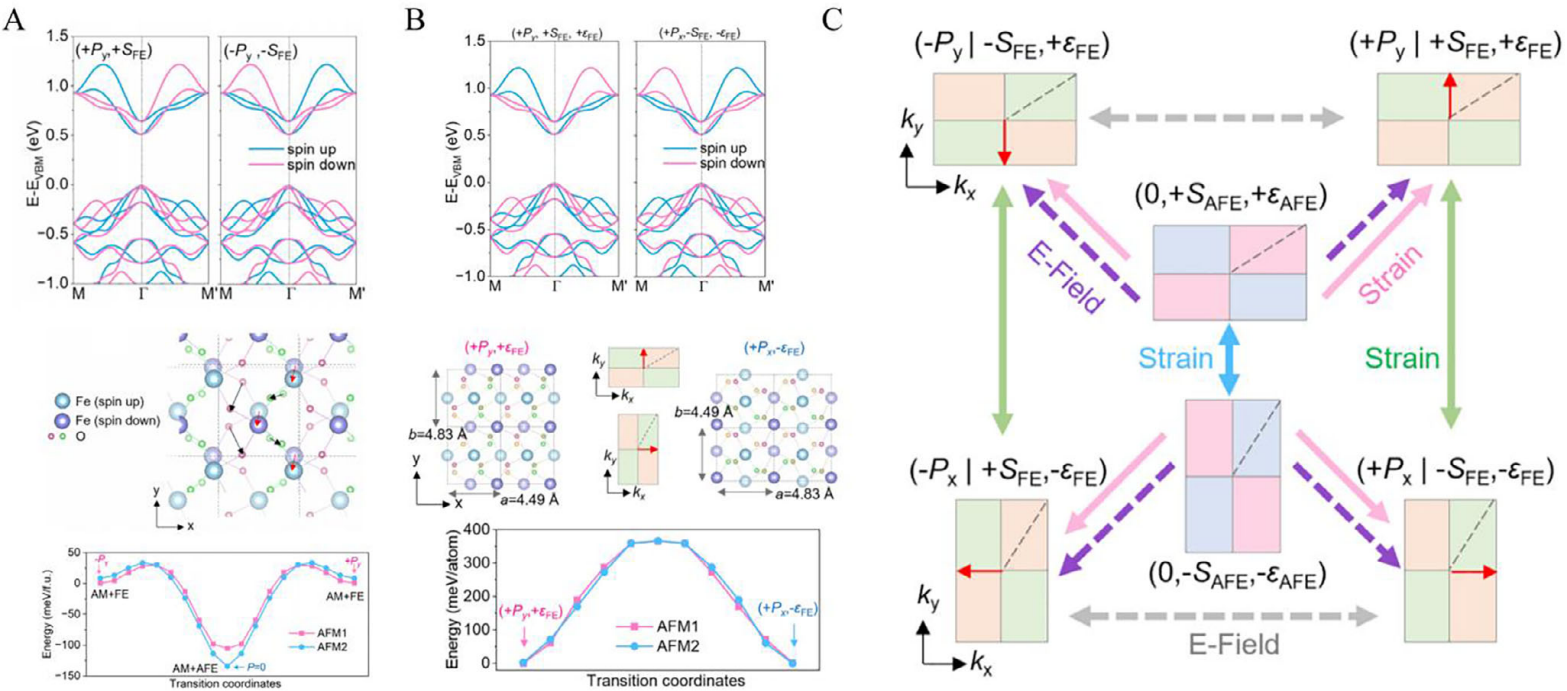
(A) Band structures of p‐FeO_2_ in the FE phases, respectively. FE transition pathways and energy barriers between FE phases in p‐FeO_2_. (B) Band structures of p‐FeO_2_ monolayer in distinct FA phases. Transition pathways and energy barriers between FA phases in p‐FeO_2_. (C) The interplay of ferroic orders in both FE and AFE phases gives rise to six switchable polarization states, which can be reversibly modulated by applying strain or electric fields. Reproduced with permission [79]. Copyright 2025, American Physical Society.

A compelling example of this emerging paradigm is found in a pentagonal FeO_2_ (p‐FeO_2_) monolayer, exhibiting intrinsic triferroicity, where AM, (A)FE, and FA orders coexist and are symmetry‐entangled [79]. In this system, the pentagonal lattice geometry breaks fourfold (*C_4z_*) rotational symmetry while retaining a glide mirror plane (*M_x_*), giving rise to an in‐plane spontaneous electric polarization. This structural asymmetry not only stabilizes a robust FE order (12.5 µC/cm^2^), but also enables reversible FA switching. Magnetically, the Fe sublattices adopt a collinear AFM arrangement, but crucially, the opposing spins are related by a twofold rotation and mirror operation—specifically {*C_2y_* |(0, 1/2, 0)} and {*M_x_*} in the FE phase and {*C_2x_* |(1/2, 1/2, 0)} and {*M_x_*} in the AFE phase. Figure 9A confirms that both FE and AFE phases host AM spin order, featuring sizable band splittings of ∼396 meV in the FE phase and ∼230 meV in the AFE phase. The corresponding magnetic transition temperatures are estimated to exceed 200 K, suggesting that these triferroic states are robust and potentially functional under near‐ambient conditions.

Owing to the strong coupling between the in‐plane polarization and the AM orders, switching the FE polarization from +*P_y_* to −*P_y_* by an external electric field drives a transition between the two collinear AFM configurations (AFM1, AFM2) and simultaneously reverses the sign of the AM spin splitting, denoted as (+*P_y_*, +*S_FE_*) to (‐*P_y_*, ‐*S_FE_*), while the intermediate state corresponds to (0, +*S_AFE_*). In parallel, application of uniaxial strain causes a FA lattice transition, which rotates the polarization vector by 90° (e.g., *P_y_* to *P_x_*), and induces a corresponding reversal of the spin splitting in the momentum space (*S_FE_*) and FA strains (*ε_FE_*). As a result, two coupled states emerge, namely (+*P_y_*, +*S_FE_*, +*ε_FE_*) and (+*P_x_*, –*S_FE_*, –*ε_FE_*), as shown in Figure 9B. Considering the interplay among multiple ferroic orders in monolayer p‐FeO_2_, a total of six distinct ferroic states emerge (Figure 9C). In the FE phase, the polarization *P* is tied to a specific combination of the *S_FE_* and the *ε_FE_*, resulting in four independent FE states. Once the polarization direction is chosen, *S_FE_* and *ε_FE_* are fixed accordingly, and any change in *S_FE_* or *ε_FE_* necessarily affects *P*, while reversing *P* inevitably modifies the corresponding *S_FE_* and *ε_FE_*. Beyond this, the two intermediate AFE states possess opposite AM spin splitting *S_AFE_* and are interconverted by FA switching, which can be triggered by in‐plane strain applied along either the x or y direction. Because the FE and AFE phases have distinct lattice constants, in‐plane strain can also transform the AFE phase into the FE phase, inducing a substantial in‐plane polarization in four possible configurations. Altogether, this triferroic coupling framework allows for deterministic and reversible control over six ferroic states in p‐FeO_2_, offering a versatile platform for multistate spintronic and logic‐in‐memory applications.

## 3 Ferroelectricity, Magnetism, and Valley Coupling

The valley degree of freedom, originating from the inequivalent energy extrema (K and K′ valleys) in momentum space, provides an additional binary index analogous to charge and spin [80]. It has emerged as a key handle for encoding and manipulating information in 2D materials. In FE 2D materials, broken inversion symmetry and finite Berry curvature allow valley polarization to be generated and electrically switched together with the FE polarization, giving rise to FE–valley coupling [81]. If intrinsic magnetism is additionally present, valley polarization may further couple to both FE and magnetic order, offering a route to FE–magnetic–valley triferroic coupling. In this class of systems, valley polarization can in principle be reversed by switching the direction of the FE polarization or the Néel vector, thereby establishing coupled FE‐valley and magnetic‐valley responses. Such coupled responses are commonly achieved through intrinsic polar magnetic lattices, interfacial proximity effects in FE/magnetic heterostructures, or sliding‐induced symmetry breaking in van der Waals bilayers. Despite their different physical origins, these mechanisms all establish coupling among FE polarization, magnetism, and valley polarization, thereby extending triferroicity beyond conventional lattice‐spin coupling. In the following, we summarize representative 2D systems with coupled FE polarization, magnetism, and valley polarization, and categorize them according to their magnetic ground states.

### 3.1 Ferroelectricity, Ferromagnetism, and Ferrovalley Coupling

**FIGURE 10:**
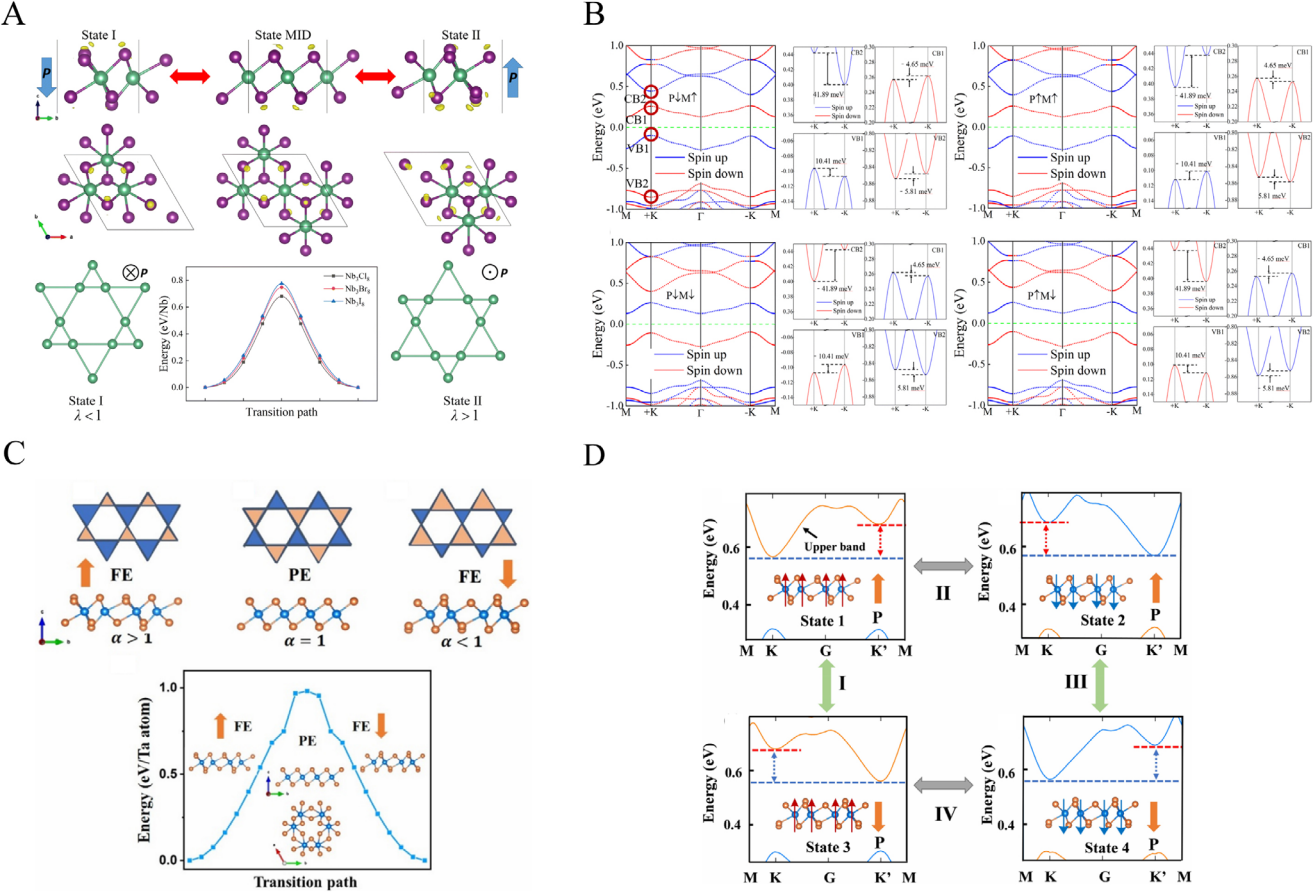
(A) Top and side views of two FE states and one paraelectric (PE) state of SL Nb_3_X_8_. FE pathway of SL Nb_3_X_8_. (B) non‐SOC and SOC band structures of SL Nb_3_X_8_ in distinct FE states. Reproduced with permission [82]. Copyright 2023, Royal Society of Chemistry. (C) Top views of two FE states and one PE state of SL Ta_3_I_8_. FE switching pathway of SL Ta_3_I_8_. (D) Spin‐resolved band structures including SOC for SL Ta_3_I_8_ in different FE states. Reproduced with permission [83]. Copyright 2024, American Chemical Society.

The Nb_3_X_8_ (X = Cl, Br, I) monolayers exhibit triferroic behavior characterized by the simultaneous presence of ferroelectricity, ferromagnetism, and ferrovalley polarization, which originates from their unique breathing Kagome lattice structure [82]. Nb_3_X_8_ monolayers exhibit a breathing Kagome network, where the Nb–Nb distances within each trimer are slightly shorter than those bridging to the clusters. This structural asymmetry breaks inversion symmetry and induces a spontaneous out‐of‐plane electric dipole moment, thereby establishing intrinsic ferroelectricity in the material, as illustrated in Figure 10A. Simultaneously, the magnetism in Nb_3_X_8_ arises from the partially filled 4d orbitals of the Nb‐trimer clusters, yielding a net magnetic moment of 1 µ_B_. Moreover, the breaking of inversion symmetries and out‐of‐plane ferromagnetism leads to spontaneous valley polarization at the ±K points in the Brillouin zone when spin–orbit coupling (SOC) is included.

To fully harness the multifunctional potential of Nb_3_X_8_ monolayers, it is essential to elucidate the coupling mechanisms among ferroelectricity, ferromagnetism, and ferrovalley polarization. This triferroic coupling is exemplified by four energetically degenerate ferroic states: P↓M↑, P↓M↓, P↑M↑, and P↑M↓. Reversing the FE polarization (P↑ to P↓) leads to a sign change in valley polarization, indicating strong FE‐valley coupling, while reversing the magnetization (M↑ to M↓) switches the spin channel of valleys due to spin–valley coupling. When both P and M are reversed, the valley splitting remains unchanged (Figure 10B). This triferroic coupling enables external control of valley polarization through either electric or magnetic fields, providing a new perspective on multistate storage.

Another similar example is proposed by Xing et al. who predicted that single‐layer Ta_3_I_8_ is an intrinsic triferroic in which ferroelectricity, ferromagnetism, and ferrovalley polarization coexist [83]. Structurally, the Ta_3_I_13_ clusters shift the centers of positive and negative charge, producing an out‐of‐plane electric dipole (Figure 10C). On the magnetic side, the Ta *d* orbitals first split into *t_2g_* and *e_g_* states under the octahedral crystal field effect and then further divide into six energy levels (1e, 1a_1_, 2e, 2a_1_, 3e, 1a_1_). Therefore, seven *d* electrons are shared within each Ta_3_ trimer, leaving a single unpaired spin in the 2a_1_ level, and yielding a magnetic moment of 1 µ_B_ per cluster. When SOC is included, the direct gap at the ±K valleys becomes asymmetric, establishing a robust spontaneous ferrovalley polarization. Furthermore, a strong coupling between three degrees of freedom is demonstrated: flipping the magnetization while maintaining the FE polarization direction causes a reversal of valley polarization, and conversely, switching the FE polarization while holding the spin ordering also inverts the valley polarization, as shown in Figure 10D. This bidirectional control confirms the existence of robust FE–valley and spin–valley coupling in monolayer Ta_3_I_8_, offering a promising pathway for valleytronic functionalities governed by external fields.

**FIGURE 11:**
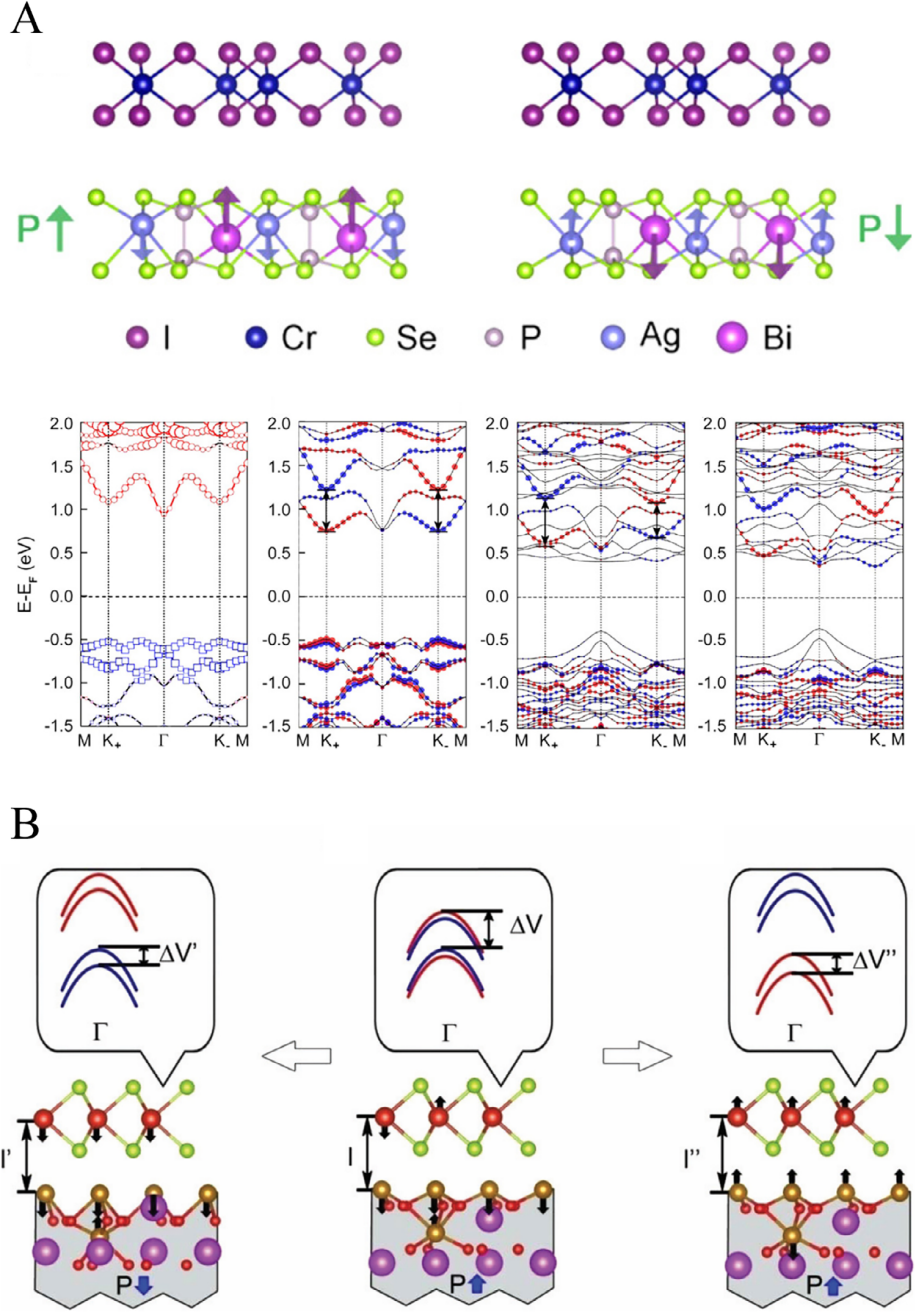
(A) Structure of AgBiP_2_Se_6_/CrI_3_ heterostructure. Band structures with and without SOC for monolayer AgBiP_2_Se_6_. Band structures with two FE AgBiP_2_Se_6_/CrI_3_ heterostructures. Reproduced with permission [84]. Copyright 2019, American Chemical Society. (B) Illustration of multiferroic control of 2H‐VSe_2_/BFO (111) heterostructure. Reproduced with permission [85]. Copyright 2019, Royal Society of Chemistry.

Interfacial engineering provides another avenue to combine distinct ferroic orders. Pei et al. proposed a van der Waals heterostructure composed of ferroelectric monolayer AgBiP_2_Se_6_ and ferromagnetic monolayer CrI_3_, thereby establishing a triferroic platform for electrically controlled valleytronic devices [84]. Monolayer AgBiP_2_Se_6_ exhibits out‐of‐plane polarization arising from antiparallel off‐centering of Ag^+^ and Bi^3+^. Owing to broken inversion symmetry and strong SOC strength of Bi, it hosts K^±^ valleys with ∼472 meV spin splitting at the conduction‐band minimum (CBM). When interfaced with monolayer CrI_3_, whose ferromagnetism is largely preserved, magnetic proximity breaks the degeneracy between K^+^ and K^−^, giving rise to a ferrovalley state as shown in Figure 11A. Moreover, the ferrovalley response is further tunable by FE switching, as the polarization direction of AgBiP_2_Se_6_ is reversed, the valley splitting is reconfigured, enabling an electrically controlled ferrovalley functionality in the AgBiP_2_Se_6_/CrI_3_ heterostructure.

Similarly, in the 2H‐VSe_2_/BiFeO_3_(111) heterostructure [85], a coupled FE–FM–ferrovalley response is achieved. BiFeO_3_ (BFO) is a room‐temperature multiferroic that hosts switchable FE polarization along [111] together with G‐type AFM order, while monolayer 2H‐VSe_2_ is a 2D ferromagnet that exhibits an intrinsic ferrovalley behavior enabled by exchange interaction among V‐3d electrons. The coupling among these orders is mediated by a tunable interfacial Fe–V exchange interaction: reversing the FE polarization direction or Fe magnetic moments of the BFO substrate modifies the interfacial Fe–V distance and exchange strength, thereby tailoring the V magnetic moment as well as the spin and valley splitting in monolayer VSe_2_, as shown in Figure 11B. This establishes a practical route toward electrically controlled valleytronic devices.

### 3.2 Ferroelectricity, Antiferromagnetism, and Valley Coupling

The emergence of sliding ferroelectricity has fundamentally enriched the conceptual landscape of multiferroicity. Moreover, the sliding‐induced control of symmetry and interlayer coupling provides an important and practical route toward realizing triferroicity in 2D systems. A concrete example is the Janus 2H‐VSeS bilayer, proposed as a semiconducting triferroic in which sliding ferroelectricity, ferrovalley, and antiferromagnetism coexist [86].

**FIGURE 12:**
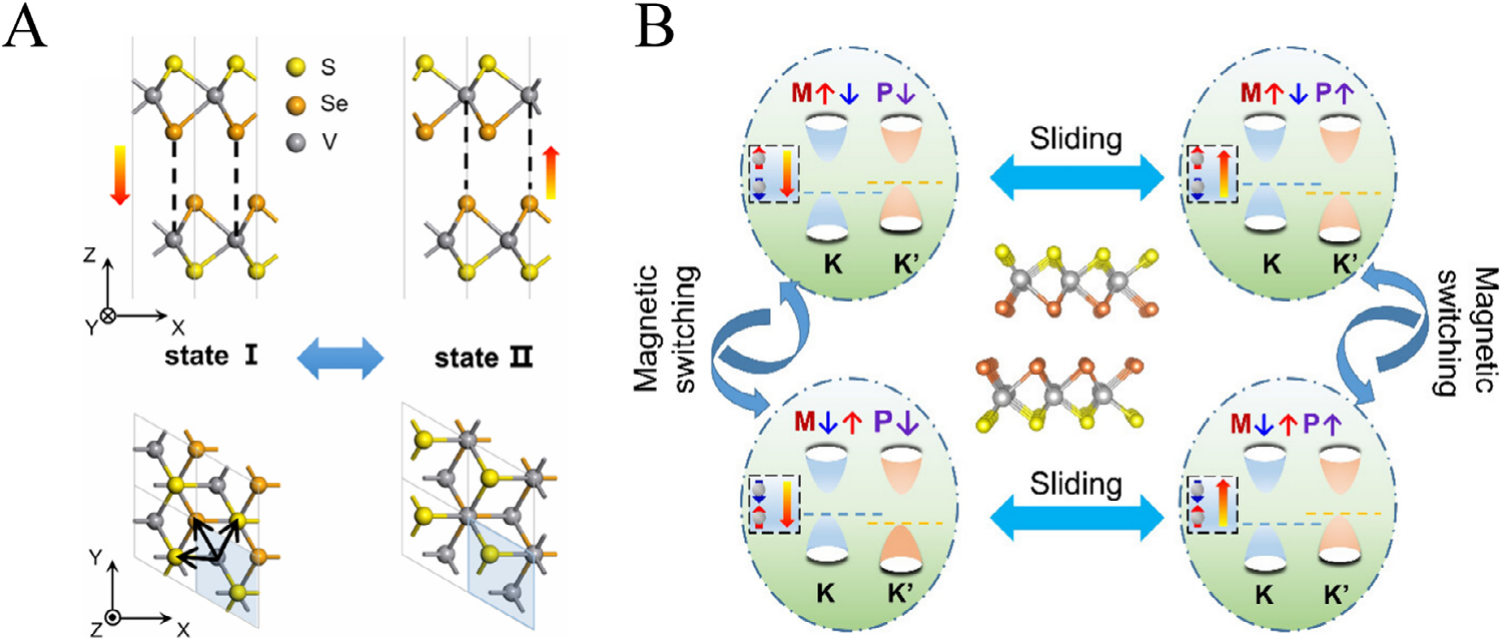
(A) Side and top views of the Janus 2H‐VSeS bilayer in a back‐to‐back stacking order. (B) Mechanism of triferroicity in 2H‐VSeS bilayer. Reproduced with permission [86]. Copyright 2024, American Chemical Society.

The ferroelectricity of Janus 2H‐VSeS bilayer originates from the back‐to‐back stacking order, which breaks inversion symmetry and induces a switchable out‐of‐plane polarization. The two degenerate FE phases, state I and state II, are displayed in Figure 12A. AFM magnetic order is stabilized by interlayer interactions, yielding a zero net magnetic moment. Ferrovalley polarization emerges as the valley degeneracy at the K and K' points is lifted due to specific stacking. To demonstrate the intrinsic triferroic coupling in Janus 2H‐VSeS bilayer, four degenerate states were constructed: P↓M↑↓, P↓M↓↑, P↑M↑↓, and P↑M↓↑. As shown in Figure 12B, valley polarization can be modulated by either sliding ferroelectricity or magnetic order, which indicates that multiple ferroic states can be switched by external electric or magnetic fields, enabling mutual control among charge, spin, and valley orders.

**FIGURE 13:**
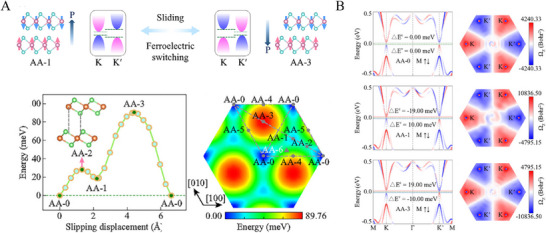
(A) Schematic of the coexisting triferroic in 2H phase bilayer OsBr_2_. Energy barriers via interlayer sliding of the 2H phase bilayer OsBr_2_. (B) Band structures and Berry curvatures of 2H AA‐0, AA‐1, AA‐3 bilayer OsBr_2_ with SOC effect. Reproduced with permission [87]. Copyright 2025, Wiley‐VCH.

Another candidate is the bilayer OsBr_2_, whose ferroic properties are governed by stacking orders [87]. Due to the triangular prism crystal fields, the *d* orbitals of the 2H phase monolayer OsBr_2_ split into one a_1g_ ($d_{z^{2}}$), two e_1_ (*d_xy_*, $d_{x^{2}-y^{2}}$), and two e_2_ (*d_xz_*, *d_yz_*). The 5*d^6^* electrons of Os fill the a_1g_, while the e_1_ and e_2_ states are half‐filled. This electronic configuration renders the 2H‐phase OsBr_2_ monolayer semiconducting and yields a magnetic moment of 4 µB per Os atom. The ferroic responses in bilayer OsBr_2_ are primarily dictated by the stacking order. For example, in the 2H phase AA‐1 stacking, it exhibits an AFM ground state together with an out‐of‐plane FE polarization. It also hosts a spontaneous valley polarization arising from the breaking of *P* and *T* symmetries. Figure 13A shows the mechanism of the 2H‐phase bilayer OsBr_2_ triferroic and the associated multiple types of valley polarization. With SOC included, sliding the stacking from AA‐0 to AA‐1 induces a valley polarization between the K and K′ points, characterized by an energy splitting of 10.00 meV in the valence band and −19.00 meV in the conduction band. The Berry curvatures at K and K′ remain opposite but become inequivalent. Further sliding to AA‐3 reverses the FE polarization and thus the valley polarization, accompanied by a reversal of the Berry curvatures as illustrated in Figure 13B. Overall, bilayer OsBr_2_ provides a stacking‐engineered triferroic platform, where interlayer sliding enables reversible and multistate control of ferroelectricity, magnetism, and valley polarization (including Berry‐curvature switching).

Triferroicity is realized in bilayer NbSi_2_N_4_ via an interlayer‐sliding mechanism. Monolayer NbSi_2_N_4_ is intrinsically FM, and produces a spontaneous valley polarization that is tied to the magnetic order. Upon forming the bilayer, interlayer sliding induces a switchable out‐of‐plane FE polarization. Meanwhile, the bilayer prefers an A‐type AFM ground state, arising from FM coupling within each layer together with weak AFM coupling between the layers. As a result, FE, AFM, and ferrovalley orders coexist in this system.

**FIGURE 14:**
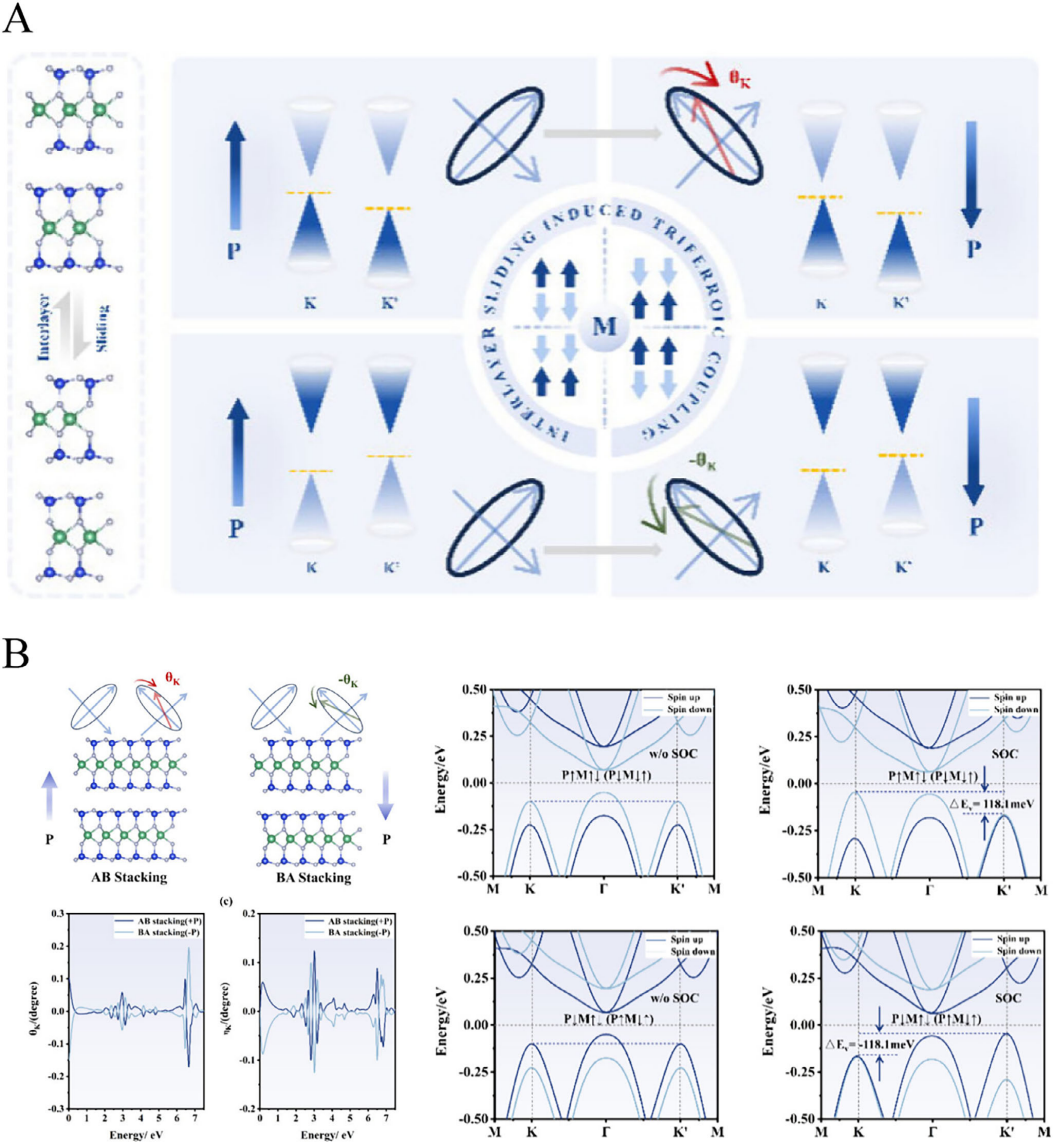
(A) Mechanism of triferroicity and magneto‐optical Kerr effect in the bilayer NbSi_2_N_4_. (B) Kerr angle and ellipticity of AB and BA stacking configurations in bilayer NbSi_2_N_4_. Spin‐resolved band structures of four degenerate states of bilayer NbSi_2_N_4_ [88]. Copyright 2025, American Chemical Society.

Building on this sliding‐induced triferroic framework, bilayer NbSi_2_N_4_ further exhibits a pronounced coupling among FE, magnetic, and valley degrees of freedom. Four degenerate stacking configurations, which are labeled as P↓M↑↓, P↓M↓↑, P↑M↑↓, and P↑M↓↑ are taken into consideration. Before considering SOC, the electronic states at the K and K′ valleys are degenerate. Upon inclusion of SOC, this degeneracy is lifted, leading to the emergence of spontaneous valley polarization. As illustrated in Figure 14B, reversing either the FE polarization or the magnetic configuration leads to a deterministic switching of valley polarization, enabling electrically and magnetically multistate control. Moreover, the concurrent breaking of time‐reversal and inversion symmetries activates a sizable magneto‐optical Kerr effect in this AFM system. Upon FE switching, both the Kerr rotation angle and Kerr ellipticity are reversed in sign due to a strong magnetoelectric coupling. Together, these features establish bilayer NbSi_2_N_4_ as a representative example in which interlayer sliding induced triferroicity and magneto‐optical response within a single 2D platform.

## 4 Corner States in 2D Intrinsic Triferroic Materials

Recently, a new class of topological insulators, known as second‐order topological insulators (SOTIs), has been proposed. In these systems, the *d*‐dimensional bulk topology is reflected in the (*d*‐2)‐dimensional boundaries, giving rise to hinge states in 3D or corner states in 2D [89, 90, 91, 92, 93]. Notably, the corner states in 2D SOTIs are both robust and readily controllable by local external fields, suggesting that the low‐energy electrons in SOTI nanodisks acquire a new degree of freedom—namely, the corner degree [94]. When integrated into triferroic systems, a variety of novel and unconventional phenomena can emerge.

**FIGURE 15:**
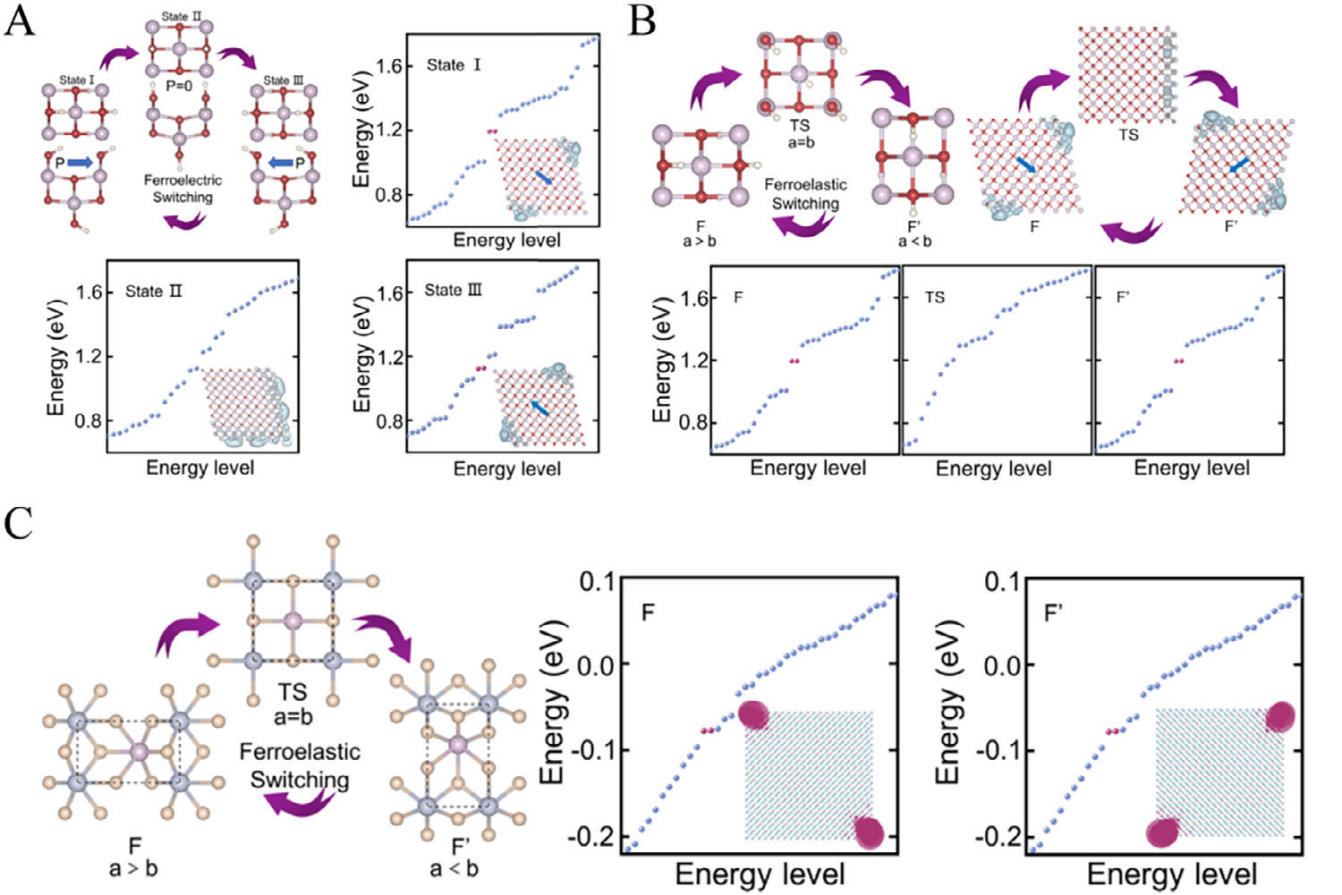
(A) Schematic diagram of FE switching in the γ‐FeO_2_H monolayer, together with the energy discrete spectra of γ‐FeO_2_H nanoflakes in the three FE states along the switching pathway. The insets represent charge distributions of corner states and/or edge states for three FE states. (B) Schematic diagram of FA switching in the γ‐FeO_2_H monolayer, together with charge distributions of corner states and/or edge states for three FA states. The energy discrete spectra of γ‐FeO_2_H nanoflakes in the three FA states along the switching pathway. (C) Schematic illustration of FA switching in the 1T′‐CrCoS_4_ monolayer, together with the energy discrete spectra of 1T′‐CrCoS_4_ nanoflakes in the two FA states along the switching pathway. The insets represent charge distributions of corner states for two FA states. Reproduced with permission [95]. Copyright 2024, American Physical Society.

A representative example of this interplay is provided by Feng et al. [95]. who realized controllable corner states in 2D intrinsic triferroic systems such as γ‐FeO_2_H and 1T′‐CrCoS_4_ monolayers. Starting with the γ‐FeO_2_H monolayer, it exhibits AFM, FE, and FA orders simultaneously. During the FE switching process, no corner state appears in the finite nanoflake at the intermediate state (state II). Upon rotating the angle between the O─H bond and the c axis, two FE states emerge; meanwhile, due to the interplay between spontaneous in‐plane polarization and Berry curvature, corner states localized at the top‐right and bottom‐left corners in FE states I and III, as shown in Figure 15A. More strikingly, because of the coexistence of FE and FA orders, an FA switching will also rotate the polarization direction. To get a better view of how the FA switching affects the corner states, a 4√2 × 4√2 γ‐FeO_2_H nanoflake is constructed (Figure 15B). In this case, the intermediate state remains nonpolar, so the charge accumulation is no longer confined to the corners but instead redistributes along the nanoflake edges. Nevertheless, the corner states in the two FA states become reversible, indicating that FA switching provides an effective means to control the corner states. Similarly, the same mechanism can be realized in 1T′‐CrCoS_4_ monolayer. As depicted in Figure 15C, during FA switching, corner states are well localized at the corners and are oriented perpendicular to the in‐plane spontaneous polarization directions. Overall, these results establish intrinsic triferroics as an ideal platform where FE and FA switching enable robust, reversible, and highly tunable control of SOTI corner states.

## 5 Summary and Perspectives

Before outlining future opportunities, it is useful to further clarify the conceptual scope of the 2D triferroic orders discussed in this Review. Here, triferroic orders are broadly defined as three coupled, controllable, and spontaneous order parameters or degrees of freedom. Based on the microscopic origin of these coupled order parameters, the 2D triferroic systems discussed above can be broadly classified into two representative categories. The first category comprises (A)FE–magnetic–FA triferroics, in which spontaneous FE polarization, magnetic orders (including FM, AFM, FIM, or AM), and FA strain are intrinsically intertwined. In these systems, FA switching directly reorients both the in‐plane polarization direction and magnetic easy axis, while FE reversal or strain modulation enables deterministic control over spin configurations. The second category corresponds to FE–magnetic–valley triferroics, where valley polarization is intrinsically locked to FE and magnetic orders. In this class, FE switching or magnetic reversal enables selective manipulation of the valley polarization, enabling multi‐state electric/magnetic control within a single system. Together, these two triferroic paradigms demonstrate how lattice, spin, and valley degrees of freedom can be synergistically integrated in 2D materials, substantially expanding the design space of multifunctional ferroic platforms beyond that of conventional multiferroics. At the same time, it should be noted that the current development of 2D triferroics remains largely theory‐driven, whereas experimental demonstrations are still relatively limited. In general, theoretical studies mainly focus on predicting candidate materials, clarifying the microscopic origin of coupled order parameters, and evaluating the feasibility of multistate switching, while experimental efforts are required to verify whether these predicted triferroic orders can be realized, stabilized, and manipulated under realistic conditions. From this perspective, the assessment of 2D triferroics should go beyond the simple coexistence of three order parameters and should further include key performance metrics, such as thermal stability, operating temperature, switching barrier, coupling strength, reversibility, readout efficiency, and device integrability.

Although the foregoing sections have systematically summarized two representative classes of triferroic couplings in 2D systems and highlighted their advantages for potential application, a longer‐term perspective suggests that this field still offers substantial room for further expansion and discovery.

First, from the perspective of microscopic mechanisms, most currently proposed 2D triferroics still rely on structural symmetry breaking to generate and couple multiple order parameters, while spin‐driven triferroicity remains comparatively rare in 2D systems. More importantly, a clear and broadly applicable microscopic design strategy has not yet been established for achieving strong intrinsic coupling among the relevant ferroic orders. Therefore, developing more general microscopic principles for realizing stronger intrinsic coupling, deterministic multistate switching remains a central challenge for the field.

Second, from the perspective of materials realization, many candidate 2D triferroics reported so far are still limited to theoretical prediction. Their experimental synthesis, structural quality, environmental stability, substrate dependence, and finite‐temperature robustness all require much more systematic investigation. Because the coupled ferroic responses in 2D systems are highly sensitive to lattice distortion, interlayer stacking, and external fields, even subtle differences in growth conditions or supporting substrates may significantly modify the predicted triferroic behavior. Therefore, bridging the gap between first‐principles design and experimentally accessible intrinsic triferroics remains one of the most important tasks for the field.

Third, from the viewpoint of device implementation, the multistate switchability already demonstrated in many proposed 2D triferroics makes them highly attractive for next‐generation multifunctional devices, including nonvolatile multistate memories, spintronic logic devices, and electrically reconfigurable information platforms. However, practical device realization will require further attention to domain stability, switching pathways, fatigue behavior, read/write schemes, and interface engineering with electrodes or substrates. In particular, how to translate theoretically predicted ferroic switching pathways into robust and reproducible device operation remains an open question that deserves more focused investigation.

Finally, given that triferroic coupling already enables multistate and switchable functionalities, an important future direction is to incorporate additional degrees of freedom into the existing triferroic framework. For example, integrating valley degrees of freedom or corner states with FE, magnetic, and FA orders within a single system may enable quadri‐ferroic or even higher‐order multiferroic couplings. In this sense, the currently proposed triferroic systems may serve not only as representative coupled ferroic platforms, but also as a foundation for developing future multifunctional materials beyond the scope of conventional multiferroics.

In conclusion, research on 2D triferroics is advancing rapidly, and recent progress already points to a wide and exciting landscape ahead. While several distinct routes have been demonstrated to couple multiple ferroic orders within a single 2D platform, the underlying design space is clearly larger, suggesting ample opportunities to uncover new materials, stronger couplings, and more versatile switchable multistate behaviors.

## Conflicts of Interest

The authors declare no conflict of interest.

## Data Availability

The authors have nothing to report.
